# Supplementary material for: An open-source framework for large-scale, flexible evaluation of biomedical text mining systems
Source: J Biomed Discov Collab. 2008 Jan 29;3:1. doi: 10.1186/1747-5333-3-1 (PMC2276192; doi:10.1186/1747-5333-3-1)
Supplement: Additional file 1 — Evaluation platform source code. The additional file contains the software evaluation framework discussed in this paper. The most current release can be downloaded from SourceForge.net [1]. See the accompanying README file for instructions on installation and use. Also included in the distribution is a collection of UIMA wrappers for some commonly used BioNLP tools and annotated corpora. [file 1747-5333-3-1-S1.zip › bionlp_uima_repository-1.0.1/docs/api/edu/uchsc/ccp/util/nlp/annotation/comparison/package-summary.html]

edu.uchsc.ccp.util.nlp.annotation.comparison (Center for Computational Pharmacology Util/UIMA API)


|  |  |  |
| --- | --- | --- |
| **Overview** \| **Package** \| Class \| **Tree** \| **Deprecated** \| **Index** \| **Help** | |  |
| **PREV PACKAGE**   **NEXT PACKAGE** | **FRAMES**    **NO FRAMES**     **All Classes** |  |


---

## Package edu.uchsc.ccp.util.nlp.annotation.comparison

| **Class Summary** | |
| --- | --- |
| **AnnotationComparator** |  |
| **DocumentLevelAnnotationCreator** | This class creates document-level annotations for a given type. |
| **EntityPartsAnnotationComparator** | This class implements the functionality of the "Protein Name Parts" comparison metric described in Olson et al...  The usefulness of this kind of comparison is limited to simple entity mentions, therefore, the mention comparison will compare the entire mention hierarchy, however, the entity parts computation will only take place on the base annotation span.    For example, if for some reason you wanted to run the entity-parts metric on a group of transport annotations, where each transport mention has complex slot mentions (transported entities, source, destination, transport participants), then this method will  find equivalent transport annotations using the SloppySpanComparator if equivalent annotations are found, then the covered text for each annotation (and in this case, just for the transport annotation, not any of its slot fillers) will be tokenized on whitespace, and the entity-parts metric will be done using those tokens. |
| **IgnoreSpanComparator** | Use this span comparator when you do not care to compare spans. |
| **SharedEndSpanComparator** | For "shared end" comparison, the spans are required to have the same end index in order to return 0. |
| **SharedStartOrEndSpanComparator** | For "shared start or end" comparison, the spans are required to have the same start or end indexes in order to return 0. |
| **SharedStartSpanComparator** | For "shared start" comparison, the spans are required to have the same starting index in order to return 0. |
| **SloppySpanComparator** | For "sloppy" comparison, the spans are required to overlap by at least one character. |
| **SpanComparator** |  |
| **StrictSpanComparator** | For "strict" comparison, the spans are required to be exactly the same in order to return 0. |

---


|  |  |  |
| --- | --- | --- |
| **Overview** \| **Package** \| Class \| **Tree** \| **Deprecated** \| **Index** \| **Help** | |  |
| **PREV PACKAGE**   **NEXT PACKAGE** | **FRAMES**    **NO FRAMES**     **All Classes** |  |


---
